# The testing effect for mediator final test cues and related final test cues in online and laboratory experiments

**DOI:** 10.1186/s40359-016-0127-2

**Published:** 2016-05-31

**Authors:** Leonora C. Coppens, Peter P. J. L. Verkoeijen, Samantha Bouwmeester, Remy M. J. P. Rikers

**Affiliations:** Department of Psychology, Erasmus University Rotterdam, P.O. Box 1738, 3000 DR Rotterdam, The Netherlands; Department of Pedagogical and Educational Sciences – Education, Utrecht University, Utrecht, The Netherlands

**Keywords:** Testing effect, Semantic mediator hypothesis, Elaborative retrieval, Replication, Mechanical Turk

## Abstract

**Background:**

The testing effect is the finding that information that is retrieved during learning is more often correctly retrieved on a final test than information that is restudied. According to the semantic mediator hypothesis the testing effect arises because retrieval practice of cue-target pairs (*mother-child*) activates semantically related mediators (*father*) more than restudying. Hence, the mediator-target (*father-child*) association should be stronger for retrieved than restudied pairs. Indeed, Carpenter (2011) found a larger testing effect when participants received mediators (*father*) than when they received target-related words (*birth*) as final test cues.

**Methods:**

The present study started as an attempt to test an alternative account of Carpenter’s results. However, it turned into a series of conceptual (Experiment 1) and direct (Experiment 2 and 3) replications conducted with online samples. The results of these online replications were compared with those of similar existing laboratory experiments through small-scale meta-analyses.

**Results:**

The results showed that (1) the magnitude of the raw mediator testing effect *advantage* is comparable for online and laboratory experiments, (2) in both online and laboratory experiments the magnitude of the raw mediator testing effect *advantage* is smaller than in Carpenter’s original experiment, and (3) the testing effect for related cues varies considerably between online experiments.

**Conclusions:**

The variability in the testing effect for related cues in online experiments could point toward moderators of the related cue short-term testing effect. The raw mediator testing effect advantage is smaller than in Carpenter’s original experiment.

## Background

Information that has been retrieved from memory is generally remembered better than information that has only been studied. This phenomenon is referred to as the testing effect. The widely investigated testing effect has proven to be a robust phenomenon as it has been demonstrated with various final memory tests, materials, and participants (see for recent reviews [1–8]).

Although the testing effect has been well established empirically, the cognitive mechanisms that contribute to the emergence of the effect are less clear. Carpenter [9] suggested that elaborative processes underlie the testing effect (see [10] for a similar account). According to her elaborative retrieval hypothesis, retrieving a target based on the cue during practice causes more elaboration than restudying the entire pair. This elaboration helps retrieval at a final memory test because it causes activation of information which is then coupled with the target, hence creating additional retrieval routes. To exemplify the proposed theoretical mechanism, consider a participant who has to learn the word pair *mother - child*. Retrieving the target when given the cue (i.e., *mother*) is more likely to lead to the activation of information associated with that cue (e.g., *love, father, diapers*) than restudying the entire word pair. As a result, the activated information is associated with the target (i.e., *child*) thereby providing additional retrieval routes to the target. As a consequence, targets from previously retrieved word pairs are more likely to be retrieved than targets from restudied word pairs: the testing effect arises.

However, Carpenter [11] noted that the elaborative retrieval hypothesis was not specific about what related information is activated during retrieval practice. To address this issue, she turned to the mediator effectiveness hypothesis put forward by Pyc and Rawson [12, 13]. Based on the mediator effectiveness hypothesis, Carpenter proposed that *semantic mediators* might be more likely to get activated during retrieval practice than during restudying (henceforth denoted as the semantic mediator hypothesis). Carpenter defined a semantic mediator as a word that according to the norms of Nelson, McEvoy, and Schreiber [14] has a strong forward association with the cue (i.e., when given the cue people will often spontaneously activate the mediator) and that is easily coupled with the target. For instance, in the word pair *mother-child,* the cue (*mother*) will elicit - at least for a vast majority of people - the word *father.* The word *father* can easily be coupled with the target *child*. Hence, *father* is a semantic mediator in case of this particular word pair. The semantic mediator hypothesis predicts that the link between the semantic mediator *father* and the target *child* will be stronger after retrieval practice than after restudying.

Carpenter [11] (Experiment 2) tested this prediction using cue-target pairs such as *mother - child*. These word pairs were studied and then restudied once or retrieved once. After a 30-min distractor task, participants received a final test with one of three cue types: the original cue, a semantic mediator or a new cue that was weakly related to the target: a related cue. The latter two are relevant for the present study. Carpenter’s results showed a testing effect in the original cue condition. Moreover, at the final test the advantage of retrieval practice over restudying was greater when participants were cued with a mediator (*father*) than when they were cued with a related cue (*birth*). Furthermore, targets from the retrieval practice condition were more often correctly produced during the final test when they were cued with mediators than when they were cued with related words. This difference in memory performance between mediator-cues and related-cues was much smaller for restudied items.

These results of Carpenter’s second experiment are important because they provide direct empirical support for a crucial assumption of the semantic mediator hypothesis: the assumption that the link between a mediator and a target is strengthened more during retrieval practice than during restudying. However, there might be an alternative explanation for the findings of Carpenter’s [11] second experiment. We noted that some of the mediators used in this study were quite strongly associated with the cue. For example, one of the word pairs was *mother – child* with the mediator *father* and the related cue *birth*. In this case, there is a strong cue-mediator association from *mother* to *father* (and no forward association from *mother* to *birth*), but the mediator *father* is also strongly associated with the original cue *mother* (.706 according to the norms of Nelson et al. [14]). Now it might be possible the larger testing effect on a mediator-cued final test (*father* - _ ) as opposed to a related word-cued final test (*birth* - _ ) was caused by mediators with strong mediator-cue associations. That is, when given the mediator *father* at the final test, participants can easily retrieve the original cue *mother*. Because it is easier to retrieve the target from the original cue after retrieval practice than after restudying (in Carpenter’s Experiment 2, final test performance after a relatively short retention interval was better for tested than for restudied items; cf. [15–17]), activation of the original cue through the mediator will facilitate retrieval of the target more after retrieval practice than after restudying. By contrast, the related final test cues in Carpenter’s experiment did not have an associative relationship with the original cues, and therefore it was harder to retrieve the original cue from a related final test cue than from a mediator final test cue. If the testing effect emerges due to a strengthened cue-target link then related final test cues are less likely to produce a testing effect than mediator final test cues. Thus, strong mediator-cue associations in Carpenter’s stimulus materials in combination with a strengthened cue-target link might explain why the testing effect was larger for mediator final test cues than for related final test cues.

**Fig. 1:**
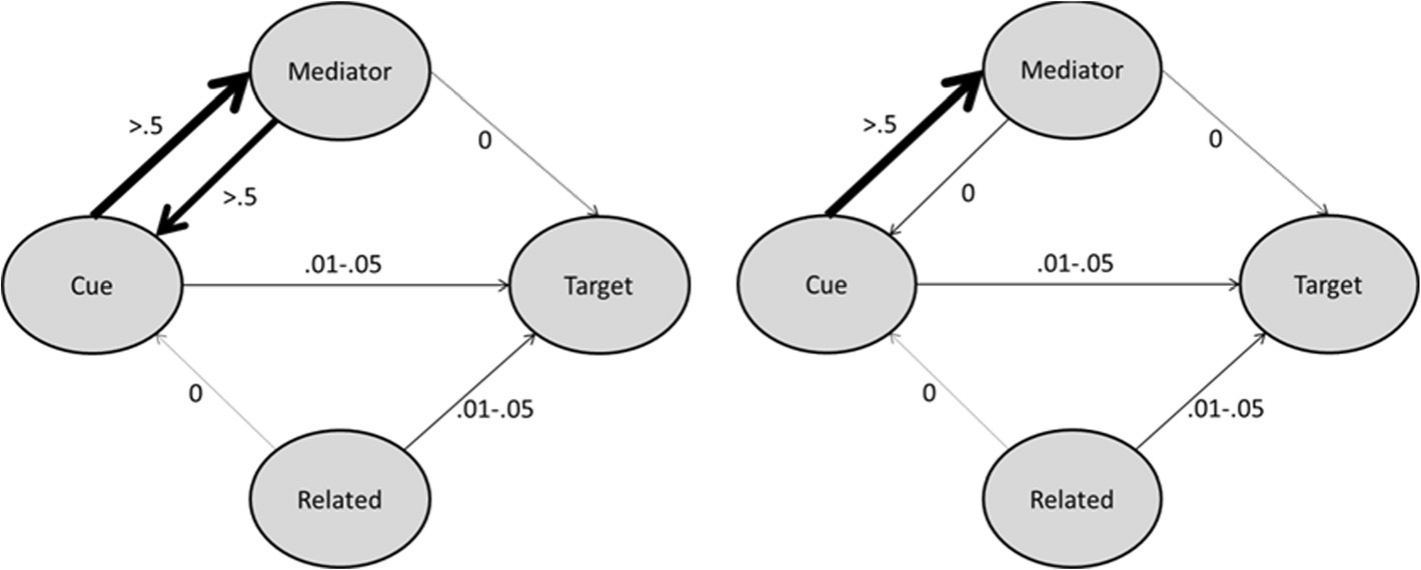
Word associations in Experiment 1. In the strong mediator-cue association condition (left), there was a strong association between the mediator and the cue. In the no mediator-cue association condition (right), there was no association between the mediator and the cue

To test this alternative explanation of the results of Carpenter’s Experiment 2, we repeated the experiment with new stimuli. We created two lists of 16 word sets that consisted of a cue, a target, a mediator, and a related cue (see Fig. 1). In both the stimuli lists, there was a weak cue-target association, a strong cue-mediator association and a weak association between the related cue and the target. The difference between the two stimuli lists was the mediator-cue association. In one stimuli list, there was a strong mediator-cue association (as illustrated in the left part of Fig. 1). This corresponds with the situation in some of the stimuli of Carpenter [11], such as *mother* – *child* with the mediator *father*. In the other stimuli list, there was no mediator-cue association (as illustrated in the right part of Fig. 1). An example of such a word set is the pair *anatomy - science* with the mediator *body*. There is no pre-existing association from *body* to *anatomy.* Therefore, if the proposed mediator *body* is not activated during learning it will not activate the original cue *anatomy* and the alternative route from the mediator through the original cue to the target is blocked.

If our alternative account is correct and the larger testing effect in the mediator-cued final test condition is caused by a strong mediator-cue association, then the stimuli with a strong mediator-cue association should yield a replication of the pattern Carpenter [11] found: a larger testing effect on a mediator-cued final test than on a related-cue-cued final test. By contrast, for stimuli without a mediator-cue association the magnitude of the testing effect should not differ between mediator final test cues and related final test cues. It should be noted that Carpenter’s semantic mediator hypothesis predicts a larger testing effect on a mediator-cued final test than on a related-cue-cued final test for both stimuli lists.

## Experiment 1

### Methods

#### Participants

For Experiment 1, we recruited participants via Amazon Mechanical Turk (MTurk; http://www.mturk.com). MTurk is an online system in which requesters can open an account and post a variety of tasks. These tasks are referred to as human intelligence tasks, or HITS. People who register as MTurk workers can take part in HITS for a monetary reward. Simcox and Fiez [18] list a number of advantages of the MTurk participants pool as compared to the (psychology) undergraduates participants pool from which samples are traditionally drawn in psychological research. First, MTurk participants are more diverse in terms of ethnicity, economic background and age, which benefits the external validity of MTurk research. Second, MTurk provides a large and stable pool of participants from which samples can be drawn year round. Third, experiments can be run very rapidly via MTurk. A disadvantage, however, is that the workers population might be more heterogeneous than the undergraduate population and that they complete the online task under less standardized conditions. This generally leads to more within subject variance which in turn - ceteris paribus - deflates the effect-size.

Participants in Carpenter’s [11] original experiment were undergraduate students instead of MTurk workers. Hence, our sample is drawn from a different population than hers. However, we think this difference is not problematic for a number of reasons. For one, nowhere in the original paper does Carpenter indicate that specific sample characteristics are required to obtain the crucial finding from her second experiment. Also, evidence is accumulating that cognitive psychological findings translate readily from the psychological laboratory to the online Mechanical Turk platform (e.g., [19–23]). In addition, replicating Carpenter’s findings with a sample from a more heterogeneous population than the relatively homogeneous undergraduate population would constitute evidence for the robustness and generality of Carpenter’s findings. This in turn would rule out that Carpenter’s findings are restricted to a specific and narrow population.

Two hundred thirty-five (235) United States residents completed the experiment via Mechanical Turk. Participants were paid $1.50 for their participation. The data of 9 participants were not included in the analysis because their native language was not English, leaving 226 participants (142 females, 84 males, age range 19–66, mean age 35.4, *SD* = 11.7). Participants were randomly assigned to conditions.

#### Materials and design

A 2 (list: strong mediator-cue association vs. no mediator-cue association) × 2 (learning condition: restudy vs. retrieval practice) × 2 (final test cue: mediator vs. related) between-subjects design was used. To investigate the effect of the mediator-cue association, we used the association norms of Nelson et al. [14] to create two lists of 16 word sets (see Appendix A). Each word set consisted of a cue and a target (weak cue-target association, .01 - .05), a mediator (strong cue-mediator association, >.5) and a related cue (weak related word-target association, .01 - .05). The difference between the two lists was the mediator-cue association. In one of the lists, the mediator-cue association in each word set was higher than .5. In the other list, the mediator-cue association in each set was 0 (see Fig. 1).

The experiment was created and run in Qualtrics [24] in order to control timing and randomization of stimuli.

#### Procedure

The procedure was identical to that of Experiment 2 of Carpenter [11] with the exception of the original cue final test condition, which we did not include because it was not relevant to the current research question. The experiment was placed as a task on MTurk with a short description of the experiment (‘this task involves learning word pairs and answering trivia questions’). When a worker was interested in completing the task, she or he could participate in the experiment by clicking on a link and visiting a website.

The welcome screen of the experiment included a description of the task and questions about participants’ age, gender, mother tongue, and level of education. In addition, participants rated three statements about the testing environment on a 5-point Likert scale. After the participant answered these questions, the learning phase began. In the learning phase all 16 cue-target pairs in one of the lists were shown in a different random order for each participant. The cue was presented on the left side of the screen and the underlined target was presented on the right. The task of the participants was to judge how related the words were on a scale from 1 to 5 (1 = not at all related – 5 = highly related), and to try to remember the word pairs for a later memory test. The study trials were self-paced. After the study trials, there was a short filler task of 30 s, which involved adding single-digit numbers that appeared on the screen in a rapid sequence. Then the cue-target pairs were presented again in a new random order during restudy or retrieval practice trials. Restudy trials were the same as study trials; participants again indicated how related the words were on a scale from 1 to 5. In retrieval practice trials, only the cue was presented and participants had to type the target in a text box to the right of the cue. Both the restudy and retrieval practice trials were self-paced, as was the case in Carpenter’s [11] Experiment 2.

After a filler task of 30 min, in which participants answered multiple-choice trivia questions (e.g., ‘What does NASA stand for? A. National Aeronautics and Space Administration; B. National Astronauts and Space Adventures; C. Nebulous Air and Starry Atmosphere; D. New Airways and Spatial Asteroids’), the final test began. Participants were informed that they would see words that were somehow related to the second, underlined word of the word pairs they saw earlier, and that their task was to think of the target word that matched the given word and enter the matching word in a text box. An example, using words that did not occur in the experiment, was included to elucidate the instructions. During the final test, participants were either cued with the mediator or with the related cue of each word pair. The cue was presented on the left side of the screen and participants entered a response into a text box on the right side of the screen. The final test was self-paced.

To end the experiment, participants rated five concluding statements about the clarity of instructions, motivation, effort, and concentration on a 5-point Likert scale. The duration of the entire experiment was about 45 min.

### Results

An alpha level of .05 was used for all statistical tests reported in this paper. Minor typing errors in which one letter was missing, added or in the wrong place were corrected before analysis.

#### Working conditions

The three statements about working conditions of the participants were rated as follows: ‘I’m in a noisy environment’: mean rating 1.5 (*SD* = 0.77), ‘There are a lot of distractions here’: mean rating 1.52 (*SD* = 0.74), ‘I’m in a busy environment’: mean rating 1.34 (*SD* = 0.66). The statements at the end of the experiment were rated as follows: ‘All instructions were clear and I was sure of what I was supposed to do’: mean rating 4.02 (*SD* = 1), ‘I found the experiment interesting’: mean rating 4.02 (*SD* = 1), ‘The experiment was difficult’: mean rating 4.06 (*SD* = 0.98), ‘I really tried to remember the word pairs’: mean rating 4.51 (*SD* = 0.79), ‘I was distracted during the experiment’: mean rating 1.83 (*SD* = 0.98).

To make sure the working conditions of the MTurk workers resembled those of participants in the laboratory as much as possible we only included those participants in the subsequent analyses who scored 1 or 2 on the last question (i.e., “I was distracted during the experiment”). The resultant sample consisted of 181 participants.

#### Intervening test

In the list with no mediator-cue associations the mean proportion of correct targets retrieved on the intervening test was .91 (*SD* = .12) in the mediator final-test condition and .84 (*SD* = .23) in the related final-test condition. In the list with strong mediator-cue associations, the mean proportion of correct targets retrieved on the intervening test was .97 (*SD* = .09) in the mediator final-test condition and .94 (*SD* = .09) in the related final-test condition.

#### Final test

The proportion of correctly recalled targets on the final test for the no mediator-cue (no MC) association list and the strong mediator-cue association list (strong MC) are presented in the second and third row of Table 1.

**Table 1:** Setting, Design, Sample Size and Results of the Experiments in the Small-Scale Meta Analyses

Experiment	Setting	Design	Total *n*	*M* testing mediator (*SD*)	*M* restudy mediator (*SD*)	*M* testing related (*SD*)	*M* restudy related (*SD*)
Coppens et al. Exp1 No-Mc	Online	2 retrieval cue (mediator vs. related) × 2 learning (restudy vs. testing) between subjects	87	0.26 (0.26)	0.13 (0.24)	0.21 (0.21)	0.16 (0.17)
Coppens et al. Exp1 Strong Mc	Online	2 retrieval cue (mediator vs. related) × 2 learning (restudy vs. testing) between subjects	94	0.50 (0.46)	0.40 (0.38)	0.38 (0.23)	0.14 (0.13)
Coppens et al. Exp2	Online	2 retrieval cue (mediator vs. related) × 2 learning (restudy vs. testing) between subjects	141	0.36 (0.31)	0.24 (0.25)	0.50 (0.27)	0.37 (0.26)
Coppens et al. Exp3	Online	2 retrieval cue (mediator vs. related) × 2 learning (restudy vs. testing) between subjects	95	0.57 (0.33)	0.29 (0.27)	0.31 (0.21)	0.32 (0.24)
Carpenter 2011 Exp2	Lab	2 retrieval cue (mediator vs. related) × 2 learning (restudy vs. testing) between subjects	40	0.58 (0.23)	0.23 (0.12)	0.29 (0.18)	0.18 (0.16)
Rawson et al. Appendix B long lag	Lab	2 retrieval cue (mediator vs. related) × 2 learning (restudy vs. testing) mixed with retrieval cue within subjects	65	0.28 (0.25)	0.15 (0.19)	0.18 (0.17)	0.11 (0.15)
Rawson et al. Appendix B short lag	Lab	2 retrieval cue (mediator vs. related) × 2 learning (restudy vs. testing) mixed with retrieval cue within subjects	63	0.28 (0.26)	0.12 (0.18)	0.15 (0.18)	0.09 (0.12)
Brennan, Cho & Neely Set A	Lab	Mediator cue only, learning (restudy vs. testing) manipulated between subjects	68	0.27 (0.20)	0.19 (0.16)		
Brennan, Cho & Neely Set B	Lab	Mediator cue only, learning (restudy vs. testing) between subjects	68	0.14 (0.12)	0.06 (0.08)		

##### No mediator-cue association

A 2 (learning condition: restudy vs. retrieval practice) × 2 (final test cue: related vs. mediator) between-subjects analysis of variance (ANOVA) on the proportion correctly recalled targets on the final test yielded a small, marginally significant main effect of learning condition, *F*(1,83) = 3.416, *p* = .068, η^*2*^_*p*_ = .040. Overall, mean target retrieval was higher for cue-target pairs learned through retrieval practice than through restudying, i.e., a testing effect. The effect of final test cue was very small and not significant, *F*(1,83) = 0.10, *p* = .919, η^*2*^_*p*_ < .01. This suggests that mean target retrieval did not differ between related final test cues and mediator final test cues. Furthermore, the Learning Condition × Final Test Cue interaction was small and not significant, *F*(1,83) = 0.875, *p* = .352, η^*2*^_*p*_ = .010. For the crucial Learning Condition × Final Test Cue interaction effect, it is also useful to look at the difference in the testing effect between mediator cues and related cues. In this case, the difference was .08 indicating that the testing effect (mean proportion correct for tested targets - mean proportion correct for restudied targets) was about 14 % points higher for mediator final test cues than for related cues. The direction of this *mediator testing effect advantage* is in line with Carpenter’s results (i.e., a larger testing effect on a mediator-cued final test than a related word-cued final test), but in her study the advantage was much larger, i.e., 23 % points.

##### Strong mediator-cue association

A 2 (learning condition: restudy vs. retrieval practice) × 2 (final test cue: related vs. mediator) between-subjects ANOVA revealed a significant small sized main effect of learning condition, *F*(1,90) = 6.330, *p* = .0104, η^*2*^_*p*_ = .066: mean target retrieval was higher for cue-target pairs learned through retrieval practice than through restudying (i.e., a testing effect). Furthermore, we found a small significant main effect of final test cue, *F*(1,90) = 8.190, *p* = .005, η^*2*^_*p*_ = .083. The mean final test performance was better for mediator final test cues than for related final test cues. The Learning Condition × Final Test Cue interaction was small and not significant, *F*(1,90) = 1.024, *p* = .314, η^*2*^_*p*_ = .011. The testing effect for mediator cues was about 14 % points *smaller* than for related cues. This mediator testing effect *disadvantage* is inconsistent with Carpenter’s [11] mediator testing effect advantage.

### Discussion

The results of Experiment 1 revealed no significant interaction effect between final test cue and learning condition in either of the two lists. The pattern of sample means showed, however, a larger testing effect for mediator final test cues than for related final test cues in the list with no mediator-cue associations. This pattern of results is similar to the one observed by Carpenter [11] in her second experiment. By contrast, in the list with strong mediator-cue associations, the testing effect was larger for related final test cues than for mediator final test cues. Taken together, these findings are not in line with the predictions based on our alternative account of the findings from Carpenter’s second experiment. Reasoning from this account, we expected to replicate Carpenter’s finding in the list with the strong mediator-cue associations. In addition, with respect to the list with no mediator-cue associations, we predicted similar testing effects for the mediator final test cues and the related final test cues. However, the findings from Experiment 1 are also inconsistent with the semantic mediator hypothesis. According to this hypothesis mediator final test cues ought to produce a larger testing effect than related final test cues both in the strong mediator-cue association list and in the no mediator-cue association list.

The outcomes of Experiment 1, which failed to corroborate the semantic mediator hypothesis, casts some doubt on the reliability of Carpenter’s [11] results. This doubt was amplified because Carpenter’s second experiment had a 2 × 2 between subjects design with only 10 participants per cell. Such a small sample is problematic because all other things being equal (i.e., alpha level, effect size and the probability of the null hypothesis being true), the probability that a significant result reflects a Type-1 error increases with a smaller sample size [25]. Consequently, it is important to assess the replicability of Carpenter’s findings. To this aim, we conducted a replication of Carpenter’s experiment, using the same procedure and learning materials.

## Experiment 2

### Methods

#### Participants

One hundred seventy-three (173) United States residents who had not participated in Experiment 1 completed the experiment via MTurk (http://www.mturk.com). Participants were randomly assigned to conditions of the factorial design mentioned below. They were paid $1.60 for their participation. Eight participants were excluded from further analysis because their native language was not English, leaving 165 participants (99 females, 66 males, age 18–67, mean age 34.6, *SD* = 12.2). Of these participants, 82 learned the word pairs through restudy and 83 learned the word pairs through retrieval practice. Forty-four participants in the restudy condition and 47 participants in the retrieval practice condition completed the final test with mediator cues. Thirty-eight participants in the restudy condition and 36 participants in the retrieval practice condition completed the final test with related cues.

#### Materials and design

We used a 2 (learning condition: restudy vs. retrieval practice) × 2 (final test condition: mediator vs. related) between-subjects design. Participants studied the same word pairs Carpenter [11] used (see Appendix B). The experiment was programmed and run in Qualtrics [24].

#### Procedure

The procedure was identical to that of Experiment 1.

### Results and discussion

#### Working conditions

The three statements about the current working environment of the participants were rated as follows: ‘I’m in a noisy environment’: mean rating 1.35 (*SD* = 0.59), ‘there are a lot of distractions here’: mean rating 1.38 (*SD* = 0.57), ‘I’m in a busy environment’: mean rating 1.32 (*SD* = 0.66). The statements at the end of the experiments were rated as follows: ‘I only participated in this experiment to earn money’: mean rating 3.25 (*SD* = 1.2), ‘I found the experiment interesting’: mean rating 3.88 (*SD* = 1.01), ‘The experiment was boring’: mean rating 2.58 (*SD* = 1.14), ‘The experiment was difficult’: mean rating 3.45 (*SD* = 1.14), ‘I really tried to remember the word pairs’: mean rating 4.71 (*SD* = 0.52), ‘I was distracted during the experiment’: mean rating 1.63 (*SD* = 0.89).

To make sure the working conditions of the MTurk workers resembled those of participants in the lab as much as possible, we only included those participants in the subsequent analyses who scored 1 or 2 on the last question (i.e., “I was distracted during the experiment”). The resultant sample consisted of 141 participants.

#### Intervening test

On the intervening test, participants correctly retrieved .89 (*SD* = .19) of the targets on average in the related final test cue condition, and .93 (*SD* = .17) in the mediator final test condition.

#### Final test

The fourth row of Table 1 shows the proportion correctly recalled targets on the final test per condition. A 2 (learning condition: restudy vs. retrieval practice) × 2 (final test cue: mediator vs. related) between-subjects ANOVA with the proportion correctly recalled final test targets as dependent variable yielded a small but significant main effect of learning condition, *F*(1,137) = 6.914, *p* = .010, η^*2*^_*p*_ = .048, indicating that final test performance was better for retrieved than restudied word pairs (i.e., a testing effect), and a small main effect of final test cue, *F*(1,137) = 8.852, *p =* .003, η^*2*^_*p*_ = .069, indicating better final test performance with related cues than with mediator cues. There was a very small non-significant Learning Condition × Final Test Cue interaction, *F*(1,137) = 0.067, *p* = .796, η^*2*^_*p*_ < .001, indicating that the effect of learning condition did not differ between final test cue conditions. Furthermore, and contrary to Carpenter’s [11] results, the testing effect for mediator cues was numerically even *smaller* than for related cues.

In sum, the results from our Experiment 2 are inconsistent with Carpenter’s [11] second experiment, and with the semantic mediator hypothesis for that matter. However, our sample was drawn from a different population than Carpenter’s sample, and although there is no reason to expect that this should matter it might be possible that the effect under interest is much smaller or even absent in the population of MTurk workers. Alternatively, it might be that there is a meaningful effect in the MTurk population but that we were unlucky enough to stumble on an extreme sample and our results reflect a Type II error. To gain insight into what happened, we aimed to assess the robustness of our findings by conducting a replication of our Experiment 2 and hence of Carpenter’s original experiment.

## Experiment 3

### Methods

#### Participants

One hundred eighteen (118) United States residents who had not participated in Experiment 1 or Experiment 2 completed the experiment via MTurk (http://www.mturk.com). Participants were randomly assigned to conditions. They were paid $1.33 for their participation. Two participants were excluded from further analysis because their native language was not English, leaving 116 participants (78 females, 38 males, age 19–67, mean age 33.4, *SD* = 11.9). Of these participants, 59 learned the word pairs through restudy and 57 learned the word pairs through retrieval practice. Thirty participants in the restudy condition and 26 participants in the retrieval practice condition completed the final test with mediator cues. Twenty-nine participants in the restudy condition and 31 participants in the retrieval practice condition completed the final test with related cues.

#### Materials, design, procedure

Materials, design, and procedure were the same as in Experiment 2.

### Results and discussion

#### Working conditions

The three statements about the current working environment of the participants were rated as follows: ‘I’m in a noisy environment’: mean rating 1.48 (*SD* = 0.74), ‘there are a lot of distractions here’: mean rating 1.44 (*SD* = 0.62), ‘I’m in a busy environment’: mean rating 1.40 (*SD* = 0.8). The statements at the end of the experiments were rated as follows: ‘I only participated in this experiment to earn money’: mean rating 3.56 (*SD* = 1.11), ‘I found the experiment interesting’: mean rating 3.79 (*SD* = 0.99), ‘The experiment was boring’: mean rating 2.85 (*SD* = 1.21), ‘The experiment was difficult’: mean rating 3.37 (*SD* = 1.11), ‘I really tried to remember the word pairs’: mean rating 4.68 (*SD* = 0.54), ‘I was distracted during the experiment’: mean rating 1.78 (*SD* = 0.99).

As in Experiment 1 and 2, we only included participants in the subsequent analyses who scored 1 or 2 on the latter question. This led to a final sample of 95 participants.

#### Intervening test

On the intervening test, participants correctly retrieved .94 (*SD* = .12) of the targets in the related final test cue condition and .95 (*SD* = .09) in the mediator final test cue condition.

#### Final test

The fifth row of Table 1 shows the proportion correctly recalled targets on the final test per condition. A 2 (learning condition: restudy vs. retrieval practice) × 2 (final test cue: mediator vs. related) between-subjects ANOVA on these proportions yielded a small significant main effect of learning condition, *F*(1,80) = 4.935, *p* = .029, η^*2*^_*p*_ = .058, indicating that final test performance was better for retrieved than restudied word pairs (i.e., a testing effect). There was a small significant main effect of final test cue, *F*(1,80) = 4.255, *p* = .042, η^*2*^_*p*_ = .051, indicating that performance was better for mediator than for related final test cues. Furthermore, there was a small significant Learning Condition × Final Test Cue interaction, *F*(1,80) = 6.606, *p* = .012, η^*2*^_*p*_ = .076, indicating that the effect of learning condition (i.e., the testing effect) was larger for mediator than for related final test cues. This pattern is consistent with Carpenter’s [11] pattern although the mediator testing effect advantage was much smaller in the current experiment than in Carpenter’s study.

### Small-scale meta-analyses

The present study resulted in four estimates of the interaction effect between learning condition (retrieval practice vs. restudy) and final test cue (mediator vs. related): two in Experiment 1, and one each in Experiments 2 and 3. The estimates of the interaction effect revealed a larger testing effect for mediator cues than for related cues in two cases (i.e., in the no-mediator-cue association list of Experiment 1, and in Experiment 3), whereas Experiment 2 and the strong mediator-cue association list in Experiment 1 demonstrated a reversed pattern. With the exception of Experiment 3, regardless of the direction, the observed interaction effects appeared to be smaller than in Carpenter’s [11] second experiment.

However, we obtained our results with MTurk participants through online experiments whereas Carpenter’s [11] original findings were obtained in the psychological laboratory with undergraduate students. To examine whether the experimental setting (MTurk/online vs. psychological laboratory) might be associated with the interaction between cue type (mediator vs. related) and the magnitude of the testing effect, we conducted two small-scale meta-analyses (see [26, 27]) in which we included the findings from Carpenter’s original study as well as findings from four highly similar unpublished experiments we were aware of (i.e., two by Rawson, Vaughn, & Carpenter [28], and two by Brennan, Cho, & Neely [29]).

The two experiments by Rawson and colleagues (see Appendix B of their paper) used Carpenter’s 16 original word pairs plus 20 new word pairs. Their experimental procedure was identical to Carpenter’s original procedure. Yet, contrary to Carpenter’s entirely between-subjects experiment, Rawson and colleagues’ experiments had a 2 Final Test Cue (mediator vs. related) × 2 Learning (restudy vs. testing) mixed design with repeated measures on the first factor.

Brennan and colleagues used two sets of materials in their experiment: Carpenter’s original materials (Set A) and a set of new materials (Set B). Participants learned both sets of materials according to Carpenter’s original procedure with restudy and retrieval practice being manipulated between subjects and with a final test involving only mediator cues.

Table 1 provides further information on the studies included in the small-scale meta-analyses as well as relevant descriptive statistics. It should be noted that all experiments in Table 1 employed extralist final test cues, i.e., cues not presented during the learning phase, which is not a standard procedure in testing effect research In addition, the final tests were always administered after a relatively short retention interval, while the testing effect usually only emerges after a long retention interval. However, apart from the related cue condition in our Experiment 3, the mean performance for items learned through testing is numerically better than the mean performance for items learned through restudy regardless of whether the final test involves mediator cues or related cues. Consequently, it seems that these extralist final test cues can reliably produce short-term testing effects. Furthermore, the standard deviations of the final test scores tend to be larger for the MTurk experiments than for the Lab experiments. To the extent that these standard deviations reflect error variance, this shows that the error variance is larger in the MTurk experiments than in the Lab experiments: a finding that does not come as a surprise given that the MTurk participants completed the experiments in less standardized settings (which leads to more unsystematic variance in final test scores) than participants in a psychological laboratory.

#### Mediator-cue testing effect

**Fig. 2:**
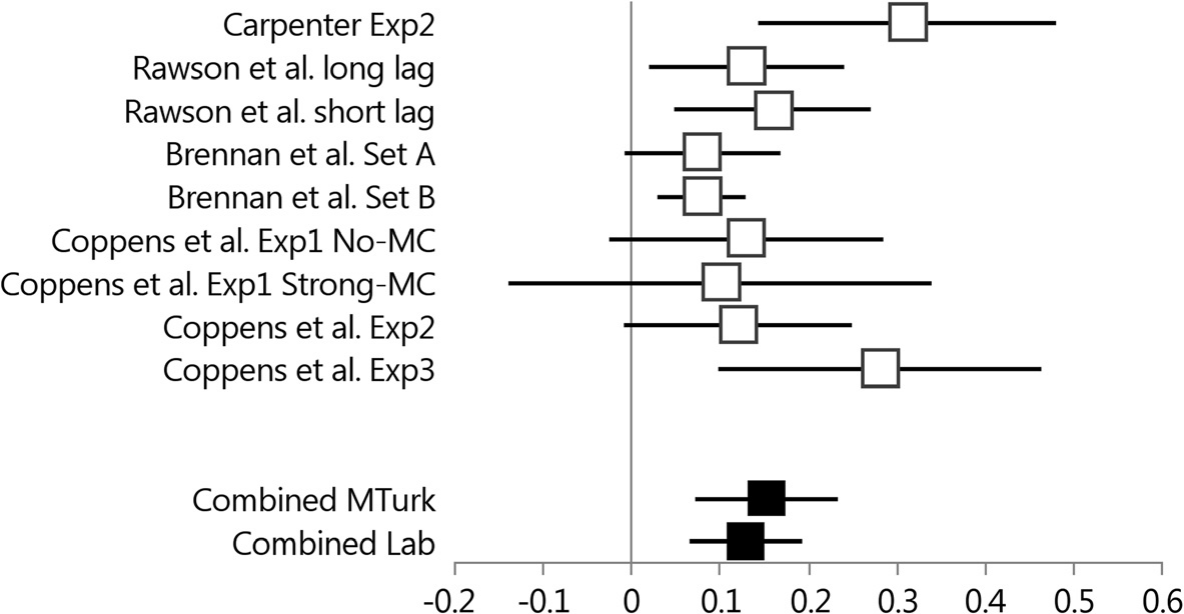
Forest plot of the 95 % confidence intervals of the mean testing advantage (final test proportion correct for tested pairs – final test proportion correct for restudied pairs) obtained with mediator final test cues for the Lab experiments (Carpenter Exp2 through Brennan et al. Set B) and the MTurk experiments (Coppens et al. Exp1 No-Mc through Coppens et al. Exp3). The combined estimates for the Lab Experiments and the MTurk experiments and the 95 % confidence intervals are also presented

Figure 2 presents the mean advantage of testing over restudying and the 95 % Confidence Interval (CI) of the mean for each experiment from Table 1 for *mediator final test cues*. Two random-effects meta-analyses were conducted to estimate the combined mean testing effect for lab experiments (i.e., estimation based on Carpenter Exp2 through Brennan et al. Set B) and for MTurk experiments (i.e., estimation based on Coppens et al.’s experiments). The estimates are presented as combined effects in Fig. 2, and they show comparable (in terms of mean difference and statistical significance) testing effects in Lab experiments (Combined *M =* 0.129, 95 % CI [0.066; 0.192]) and in MTurk experiments (Combined *M =* 0.153, 95 % CI [0.073; 0.232]. However, the estimation accuracy (width of the CI) is somewhat higher in the Lab experiments than in MTurk. Furthermore, the heterogeneity index *Q* indicates that the variance in the four MTurk testing effects can be attributed to sampling error, *Q*(3) = 2.520, *p =* .471. By contrast, the five Lab testing effects showed some heterogeneity, *Q*(4) = 9.004, *p* = .06, suggesting that the samples might have been drawn from populations with different mean testing effects. However, these heterogeneity indices should be considered with extreme caution because they are based on a very small sample of studies.

#### Related cue testing effect

**Fig. 3:**
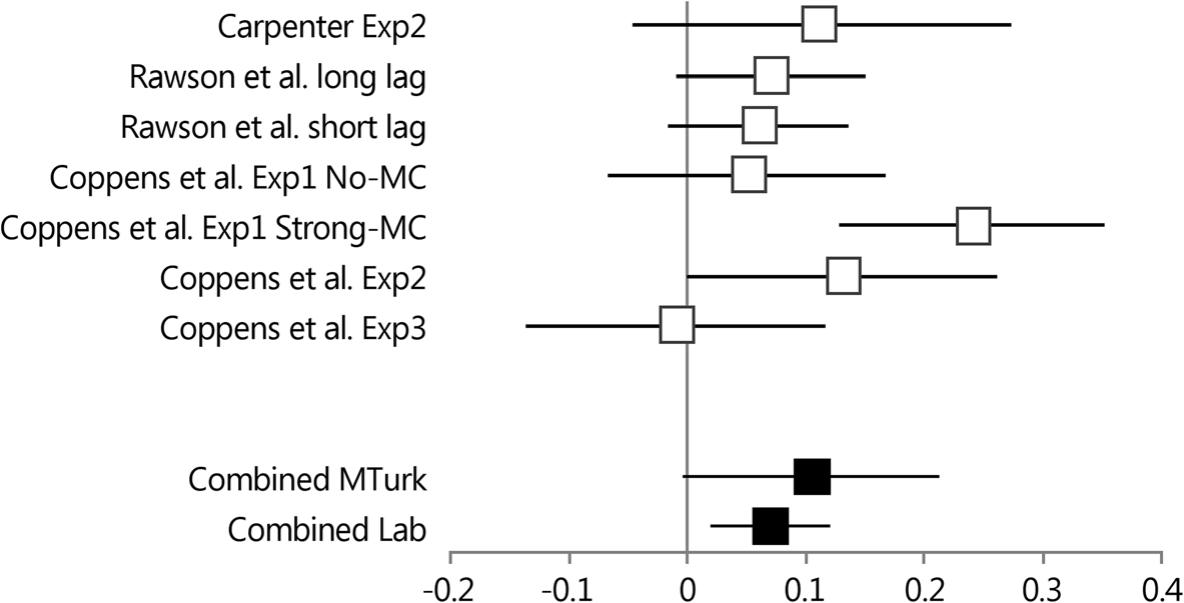
Forest plot of the 95 % confidence intervals of the mean testing advantage (final test proportion correct for tested pairs – final test proportion correct for restudied pairs) obtained with related final test cues for the Lab experiments (Carpenter Exp2 through Rawson et al. Exp2) and the MTurk experiments (Coppens et al. Exp1 No-Mc through Coppens et al. Exp3). The combined estimates for the Lab Experiments and the MTurk experiments and the 95 % confidence intervals are also presented

Figure 3 presents the mean advantage of testing over restudying and the 95 % Confidence Interval (CI) of the mean for each experiment from Table 1 for *related final test cues*. The two random-effects meta-analyses suggest that (marginally) significant testing effects can be found in Lab experiments (Combined *M =* 0.070, 95 % CI [0.019; 0.121]) and in MTurk experiments (Combined *M =* 0.105, 95 % CI [−0.005; 0.213]. However, the combined testing effect estimate is somewhat smaller and much more accurate (i.e., a narrower CI) in Lab experiments than in MTurk experiments. Also, there is a clear indication of heterogeneity for the MTurk testing effects, *Q*(3) = 10.209, *p =* .017, but not for the Lab testing effects, *Q*(2) < 1, *p =* .824. Again due to the small number of involved studies, these heterogeneity indices should be considered with extreme caution.

The combined means from the small-scale meta-analyses demonstrate that the short-term testing effect is larger for mediator cues than for related cues both in MTurk experiments (combined mediator cue testing effect = 0.153; combined related cue testing effect = 0.105) and in Lab experiments (combined mediator cue testing effect = 0.129; combined related cue testing effect = 0.070). Furthermore, the mediator testing effect *advantage* is about 5 % points in MTurk experiments and in Lab experiments. However, the testing effect for related cues appears to vary substantially across MTurk experiments and this makes it more difficult to find a Learning (restudy vs. retrieval practice) × Final Test Cue (mediator vs. related) interaction effect.

## General discussion

### Direct association hypothesis

Recently, Carpenter [11] proposed that when people learn cue-target (C-T) pairs they are more likely to activate semantic mediators (M) during retrieval practice than during restudy. In turn, due to this mediator activation, retrieval practice is assumed to strengthen the M-T link more than restudying. Hence, if people receive mediator cues during the final test, the probability of coming up with the correct target will be higher following retrieval practice than following restudy. Also, this testing effect will be smaller when related words are used as cues during the final test, which were presumably not activated during retrieval practice. Consistent with these predictions, Carpenter found in her second experiment that the testing effect was indeed larger for mediator cues than for related cues.

However, it might be possible that retrieval practice does in fact not strengthen the M-T link but only the C-T link. Now, if there is also a strong pre-existing association from the mediator to the cue, people will be able to reinstate the original cue (C) on the basis of a mediator final test cue. Subsequently, if retrieval practice strengthens the C-T link more than restudying, the use of mediator final test cues will result in a testing effect. Furthermore, the testing effect will be smaller with related final test cues that have no (or a much smaller) pre-existing association to the original cue. This line of reasoning, which Brennan, Cho and Neely [29] dubbed the *direct association hypothesis*, may provide an alternative account of the findings from Carpenter’s [11] second experiment because for some of her materials there were strong mediator-cue associations. To assess our alternative explanation of Carpenter’s findings, we replicated Carpenter’s design using cue-target pairs with no mediator-cue association (No-MC List) and cue-target pairs with strong mediator-cue associations (Strong-MC List). If Carpenter’s findings arose through mediator-cue associations, her pattern of results should emerge in the Strong-MC List but not in the No-MC List. However, the results from our Experiment 1 were not in line with these predictions. In the No-MC list, we found an interaction effect that was much smaller, but similar to the effect Carpenter found, with the testing effect being larger for mediator cues than for related cues. By contrast, in the Strong-MC list, the magnitude of the testing effect was comparable for mediator and related final test cues. Hence, the findings from Experiment 1 failed to corroborate the direct association hypothesis (see also [29]).

### Direct replication attempts

We did not find empirical evidence for our alternative explanation of Carpenter’s [11] result. However, our results were also not consistent with the semantic mediator account, which predicts a larger testing effect for mediator than for related final test cues *for both lists*. Because our findings were not consistent with this prediction, we followed up on Experiment 1 with two direct replications of Carpenter’s second experiment. Before we discuss the outcomes of our experiments, we will address the power of our experiments as well as the degree of similarity between our experiments and the original one.

An important requirement for replications (but ironically not – or hardly ever – for original studies) is that they are performed with adequate power. To determine the sample size associated with an adequate power level, one needs to know the minimal effect size in the population that is assumed to be theoretically relevant. However, in psychological research, such an effect size is almost never provided. Carpenter’s experiment is a point in case because neither the expected sizes of the two main effects (in a factorial ANOVA these effects are important since they determine in part the power associated with the interaction effect) nor the expected size of the crucial interaction effect were specified. Therefore, replicators often use the effect size in the original study for their power calculations. However, this is problematic because due to publication bias reported effect sizes are likely to overestimate the true effect size in the population (e.g., [30]). For example, in Carpenter’s original experiment almost 50 % of the variance in the dependent variable was accounted for by the linear model with the two main effects and the interaction. This effect is extraordinarily large even for laboratory research.

Given the problems associated with determining the theoretically relevant minimal effect size, Simonsohn [31] proposed to infer it from the original study’s sample size. The assumption is the original researcher(s) drew their sample to have at least some probability to detect an effect if there is actually an effect in the population. Simonsohn suggests – but he admits this is arbitrary – that the intended power of studies was at least 33 %. If we assume the original study had an intended power of 33 %, and given the original study’s sample size *n*, it is possible to determine the minimally relevant effect size. Simonsohn denotes this effect size as d33%. A replication should be powerful enough to allow for an informative failure; this means it should be able to demonstrate that the effect of interest is smaller than the minimally relevant effect size d33%. Simonsohn shows through a mathematical derivation that the required *n* “to make the replication be powered at 80 % to conclude it informatively failed, if the true effect being studied does not exist” (page 16 of the supplement; [31]) is approximately 2.5 times the original sample size. Therefore, a replication attempt of Carpenter’s [11] second experiment would require at least 2.5*40 = 100 participants. Experiment 2 and Experiment 3 of the present study had respectively 141 and 95 participants, so they met Simonsohn’s criterion for an adequately powered study.

The present experiments were set up as direct replications meaning that we tried to reinstate the methods and materials of the original experiment as closely as possible. However, there are always differences between an original experiment and a replication, even when the original researcher carries out the replication. An important question in the evaluation of replication attempts is whether existing differences render a replication uninformative regarding the reproducibility of the original results. In our view, the answer to this question depends on the strengths of the theoretical and/or practical arguments as to why the differences should matter. With respect to our experiments, one might note that testing participants online is problematic because it increases the unsystematic variance as compared to testing participants in the psychological laboratory. However, if more unsystematic variance is the only problem – implying that the raw effect of interest is the same online as in the laboratory – then it can be easily resolved by testing more participants than in the original study. We reasoned that a direct replication in addition to the original materials and procedure would require English-speaking participants who are not distracted while doing the task. Our experiments meet these criteria at least if we assume we can trust participants’ self-reports on their native language and on the conditions under which they did the experiment (another way to possibly reduce the variability would be to exclude participants based on for example catch trials or variability of response latencies, which unfortunately was not possible with our data because we did not include catch trials and could not reliably measure response latencies). Nevertheless, other researchers might hold other criteria for evaluating the comparability between our experiments and the original. The easiest way to resolve issues pertaining to comparability is to require researchers to *argue* (and not simply report without elaboration) in their papers for a range of tolerances on the method and sample parameters of their experiments. The more restrictive they are, the more they reduce the generality and scope – and consequently the interest – of their claims. Hence, researchers would be encouraged to be as liberal as possible in their methods parameters in order to increase the generality of their effect. Furthermore, if researchers routinely specify a range of allowable method and sample parameters it would become very easy to determine whether a direct replication attempt would qualify as such.

Thus, the direct replications of Carpenter’s [11] experiment, i.e., our Experiments 2 and 3 were adequately powered and in our view they should be considered as methodologically valid direct replications attempts. The results of the experiments were mixed. Experiment 2 was largely inconsistent with Carpenter’s original experiment whereas Experiment 3 clearly reinforced Carpenter’s findings. It is not clear however whether the inter-experiment variability reflects the operation of an unknown moderator to the interaction effect or whether the sample was extreme in one of the experiments (or in both but that would be unlikely).

### Small-scale meta-analyses

Taken together, the results of the present series of experiments were mixed. We found patterns similar to the results of Carpenter [11] in the No-MC list of Experiment 1 and in Experiment 3, but not in the Strong-MC list of Experiment 1 and in Experiment 2. However, our experiments were conducted online with MTurk participants, whereas Carpenter tested undergraduate psychology students in the laboratory. To examine whether this might have yielded different outcomes, we used small-scale meta-analyses to calculate combined estimates of the mean testing effect for related cues and for mediator cues both in online experiments (i.e., the four experiments from the present study) and laboratory experiments (i.e., Carpenter original experiment and four similar experiments). The outcomes of these analyses consistently revealed short term testing effects for mediator cues and related test cues. More important, however, was the finding that the mediator testing effect advantage is about 5 % points in both online experiments and in laboratory experiments. Hence, the raw mediator testing effect advantage is highly similar in online and laboratory settings. It should be noted though that this raw advantage is much smaller than in Carpenter’s original experiment, which revealed a mediator testing effect advantage of 23 % points.

In addition, we found that the mean testing effect for related cues varied considerably across online experiments, but much less across laboratory experiments. As a result, it may be more difficult to find mediator testing advantages in online experiments than in laboratory experiments. Further research needs to be conducted to assess whether the related-cue testing effect variability reflects regular random sample fluctuation or the operation of moderators. Should the latter be the case, this will either spur the further development of the semantic mediator hypothesis of the testing effect or it might lead to the refutation of the hypothesis in favor of an alternative (e.g., [4, 32, 33]).

## Conclusions

The experiments in the present study can be seen as conceptual (Experiment 1) and exact (Experiments 2 and 3) replications of Carpenter’s [11] original experiment. Recently, replication of results from psychological research has received a lot of attention (e.g., [34]) and most researchers would probably agree that replications are important. However, replication attempts are scarce and if they are performed, they are hard to publish [35, 36]. This is unfortunate, because replications inform researchers in a field about the extent to which a finding remains stable across similar experiments [35]. The current paper does exactly that and the tentative conclusions are that (1) related cues and mediator cues produce reliable short-term testing effects, (2) the magnitude of the raw mediator testing effect *advantage* is comparable for online and laboratory experiments, (3) in both online and laboratory experiments the magnitude of the raw mediator testing effect *advantage* is smaller than in Carpenter’s [11] original experiment and (4) the testing effect for related cues varies considerably between online experiments. This variability might be theoretically relevant if it points towards moderators of the related cue short-term testing effect. Furthermore, the findings of the present study are methodologically relevant to researchers who aim to build on Carpenter’s original findings: when designing their experiments, they should keep in mind that the raw mediator testing effect advantage is much smaller than in Carpenter’s experiment and that the mediator testing effect advantage may vary particularly in online samples.

### Ethics approval and consent to participate

The following ethics statement applies to all experiments in the present study. In Dutch legislation the law on medical-scientific research on humans (Wet Medisch Wetenschappelijk Onderzoek met mensen; WMO) protects people from maltreatment and experimentation. The WMO applies to research in which people are submitted to a medical or physical intervention, or to research in which a certain mode of behavior is imposed on people. According to the WMO, approval from an ethics committee is not required for certain strictly behavioral studies (note that it is almost always required for studies involving a medical or physical intervention).

We consulted the chair of the Ethics Committee Psychology of the Erasmus University Rotterdam, the Netherlands, to determine whether a formal approval of the current study was required. She concluded that a formal approval by the Ethics Committee was not necessary because the procedure was noninvasive, participants were given full disclosure of the experimental procedure, they received a payment proportionate to the task at hand, and the results of the experiments were analyzed anonymously.

The participants in all experiments were United States citizens who voluntarily subscribed for online participation in the described experiments. We did not obtain written informed consent from the participants.

### Availability of data and materials

The datasets supporting the conclusions of this article are available in the Open Science Framework repository https://osf.io/dxwz4/.

The materials used in the experiments described in this article are included within the article (and its Appendix A and Appendix B).

## Appendix A

**Table 2:** Stimuli used in Experiment 1

No mediator-cue association							
Cue	Target	Mediator	Related	C-T	C-M	M-C	R-T
Blackboard	Class	Chalk	Bored	0,014	0,676	0,000	0,048
Racquet	Sport	Ball	Coach	0,020	0,689	0,000	0,047
Architecture	Design	Building	Decoration	0,027	0,510	0,000	0,041
Mare	Night	Horse	Flashlight	0,021	0,740	0,000	0,041
Anatomy	Science	Body	Geology	0,041	0,607	0,000	0,047
Sap	Sticky	Tree	Goo	0,027	0,703	0,000	0,046
Publisher	Newspaper	Book	Horoscope	0,020	0,533	0,000	0,035
Herd	Group	Cow	Peer	0,021	0,562	0,000	0,039
Perch	Stand	Bird	Position	0,020	0,547	0,000	0,045
Oar	Man	Boat	Post	0,014	0,695	0,000	0,041
Budget	Plan	Money	Procedure	0,021	0,541	0,000	0,031
Lumber	Yard	Wood	Rake	0,040	0,596	0,000	0,041
Calories	Burn	Fat	Rope	0,040	0,527	0,000	0,039
Cork	Stopper	Wine	Rubber	0,020	0,517	0,000	0,014
Skunk	Stripe	Smell	Solid	0,016	0,559	0,000	0,028
Cradle	Rock	Baby	Sway	0,048	0,678	0,000	0,054
Strong mediator-cue association							
Cue	Target	Mediator	Related	C-T	C-M	M-C	R-T
West	Wild	East	Adventurous	0,031	0,780	0,886	0,049
Dog	Friend	Cat	Advice	0,019	0,667	0,513	0,036
Mother	Child	Father	Birth	0,010	0,597	0,706	0,015
Night	Moon	Day	Gravity	0,019	0,686	0,819	0,042
Answer	Right	Question	Incorrect	0,040	0,540	0,767	0,040
Queen	Bee	King	Insect	0,041	0,730	0,772	0,039
Bottom	Barrel	Top	Keg	0,014	0,507	0,696	0,030
Noun	Thing	Verb	Material	0,016	0,690	0,642	0,041
Front	Face	Back	Mirror	0,014	0,520	0,715	0,047
Supper	Time	Dinner	Place	0,049	0,545	0,535	0,035
Hammer	Saw	Nail	Sandpaper	0,028	0,800	0,622	0,021
Pepper	Sneeze	Salt	Sniff	0,041	0,695	0,701	0,026
Today	Show	Tomorrow	Stage	0,013	0,503	0,527	0,047
Leg	Walk	Arm	Trot	0,036	0,503	0,673	0,048
Loser	Sore	Winner	Ulcer	0,030	0,508	0,600	0,040
Volcano	Mountain	Erupt	Waterfall	0,022	0,525	0,641	0,047

*Note*. C-T indicates cue-to-target association strength, C-M indicates cue-to-mediator association strength, M-C indicates mediator-to-cue association strength, and R-T indicates related-to-target association strength. Mediator-to-target association strength and related-to-cue association strength was always 0

## Appendix B

**Table 3:** Stimuli used in Experiments 2 and 3

Cue	Target	Mediator	Related	C-T	C-M	M-C	R-T
Weapon	Knife	Gun	Ax	0,075	0,592	0,024	0,046
Coffee	Table	Tea	Banquet	0,020	0,442	0,369	0,020
Mother	Child	Father	Birth	0,010	0,597	0,706	0,015
Soil	Earth	Dirt	Continent	0,040	0,717	0,055	0,041
Sonnet	Music	Poem	Dancer	0,059	0,471	0,020	0,052
Sea	River	Ocean	Flood	0,017	0,456	0,291	0,020
Employment	Office	Job	Government	0,020	0,605	0,016	0,024
Jacket	Shirt	Coat	Hanger	0,013	0,564	0,176	0,014
Prescription	Doctor	Drug	Hospital	0,034	0,477	0,020	0,027
Trash	Paper	Garbage	Ink	0,013	0,526	0,456	0,013
Donor	Heart	Blood	Liver	0,042	0,524	0,067	0,041
Dusk	Evening	Dawn	Morning	0,042	0,609	0,454	0,047
Breeze	Summer	Wind	Mosquito	0,012	0,606	0,122	0,014
Pedestrian	Street	Walk	Neighborhood	0,032	0,597	0,000	0,034
Frame	Window	Picture	Shingle	0,014	0,811	0,316	0,014
Vocabulary	School	Words	Text	0,013	0,507	0,034	0,013

*Note*. C-T indicates cue-to-target association strength, C-M indicates cue-to-mediator association strength, M-C indicates mediator-to-cue association strength, and R-T indicates related-to-target association strength. Mediator-to-target association strength and related-to-cue association strength was always 0

## Abbreviations

C-T: cue-target
M-C: mediator-cue
M-T: mediator-target
MTurk: Amazon Mechanical Turk
